# Insulin-induced Effects on the Subcellular Localization of AKT1, AKT2 and AS160 in Rat Skeletal Muscle

**DOI:** 10.1038/srep39230

**Published:** 2016-12-14

**Authors:** Xiaohua Zheng, Gregory D. Cartee

**Affiliations:** 1 Muscle Biology Laboratory, School of Kinesiology, University of Michigan, Ann Arbor, MI, USA; 2 Department of Molecular and Integrative Physiology, University of Michigan, Ann Arbor, MI, USA; 3 Institute of Gerontology, University of Michigan, Ann Arbor, MI, USA.

## Abstract

AKT1 and AKT2, the AKT isoforms that are highly expressed in skeletal muscle, have distinct and overlapping functions, with AKT2 more important for insulin-stimulated glucose metabolism. In adipocytes, AKT2 versus AKT1 has greater susceptibility for insulin-mediated redistribution from cytosolic to membrane localization, and insulin also causes subcellular redistribution of AKT Substrate of 160 kDa (AS160), an AKT2 substrate and crucial mediator of insulin-stimulated glucose transport. Although skeletal muscle is the major tissue for insulin-mediated glucose disposal, little is known about AKT1, AKT2 or AS160 subcellular localization in skeletal muscle. The major aim of this study was to determine insulin’s effects on the subcellular localization and phosphorylation of AKT1, AKT2 and AS160 in skeletal muscle. Rat skeletal muscles were incubated *ex vivo* ± insulin, and differential centrifugation was used to isolate cytosolic and membrane fractions. The results revealed that: 1) insulin increased muscle membrane localization of AKT2, but not AKT1; 2) insulin increased AKT2 phosphorylation in the cytosol and membrane fractions; 3) insulin increased AS160 localization to the cytosol and membranes; and 4) insulin increased AS160 phosphorylation in the cytosol, but not membranes. These results demonstrate distinctive insulin effects on the subcellular redistribution of AKT2 and its substrate AS160 in skeletal muscle.

AKT, also known as protein kinase B (PKB), is a serine/threonine protein kinase with multiple regulatory functions, including the control of cell growth, survival, apoptosis, proliferation, angiogenesis and the metabolism of carbohydrate, lipid and protein^1^,^2^. Three AKT isoforms (AKT1, AKT2 and AKT3) are encoded by three distinct genes in mammalian cells^3^. AKT1 is ubiquitously expressed, and AKT2 is widely expressed, including high expression in tissues responsive to insulin-stimulated glucose transport, e.g., skeletal muscle and adipose tissue^4^,^5^. AKT3 is selectively expressed, with high expression in the brain, lung and testis, and low expression in skeletal muscle^6^,^7^. The three AKT isoforms share high homology in the N-terminal pleckstrin homology domain (PH domain; ~80%), catalytic domain (~90%) and C-terminal regulatory domain (~70–80%), but the linker region between the PH and catalytic domains is less similar (~40–50% homology)^8^.

In the insulin signaling pathway, insulin stimulation leads to the activation of phosphatidyl-inositol-3 kinase (PI3K), which in turn triggers phosphorylation of membrane phosphatidyl-inositol (PI) 4,5-bisphosphate to generate PI-3,4,5-trisphosphate (PIP3). The PH-domain of AKT can bind to PIP3, facilitating the subsequent phosphorylation of AKT on specific threonine (Thr) and serine (Ser) residues in AKT1 (Thr308 and Ser473), AKT2 (Thr309 and Ser473) and AKT3 (Thr305 and Ser472). Although insulin can induce greater phosphorylation and activation of each of the AKT isoforms, research with isoform-selective knockout mice has indicated that only AKT2 is essential for normal glycemia^9^,^10^,^11^. Furthermore, experiments using genetically modified cells and muscles have demonstrated that AKT2 is the most important AKT isoform for insulin-stimulated glucose transport^12^,^13^,^14^.

The mechanisms for AKT isoform-specific regulation of insulin-stimulated glucose transport are not fully understood. However, Gonzalez and McGraw recently provided compelling evidence that in 3T3-L1 adipocytes the isoform specificity involves AKT2’s greater susceptibility to insulin-mediated redistribution to the plasma membrane^13^. Although skeletal muscle is the tissue accounting for most of insulin’s effects on blood glucose disposal^15^, very little is known about AKT isoform-selective subcellular localization in skeletal muscle. Therefore, the first aim of the current study was to assess the influence of insulin on the subcellular localization and phosphorylation of AKT1 and AKT2 in skeletal muscle.

Among the dozens of known protein substrates of AKT^3^, AKT substrate of 160 kDa (AS160, also known as TBC1D4) has been most convincingly linked to insulin-stimulated glucose transport^16^,^17^,^18^. AS160 is a Rab-GTPase activating protein that has multiple insulin-regulatable AKT phosphomotifs, including Thr642^19^. Mutation of Thr642 to alanine (Ala) prevents phosphorylation of this site and attenuates the insulin-stimulated glucose uptake of adipocytes and skeletal muscle^17^,^18^. Prior research has also indicated that insulin can alter AS160’s subcellular localization in 3T3-L1 adipocytes^13^,^20^,^21^, but insulin’s effects on AS160 localization in skeletal muscle have not been reported. Accordingly, the second aim of the current study was to determine the effect of insulin on AS160’s subcellular localization and phosphorylation in skeletal muscle.

A variety of experimental approaches have been used to evaluate insulin’s effects on the subcellular localization of AKT isoforms in cells and tissues^13^,^22^,^23^. Some studies have relied on specialized microscopy and others have used complex differential centrifugation methods for cellular or tissue fractionation. For this study, we used a simple and rapid protocol for separating cytosolic and membrane fractions from small amounts (~70 mg) of rat skeletal muscles. The muscles were treated with a range of insulin concentrations as well as the PI3K inhibitor wortmannin to gain insights into the processes that regulate AKT1, AKT2 and AS160 phosphorylation and subcellular localization.

## Results

### Purity of the cytosolic and membrane fractions established using protein markers

**Figure 1:**
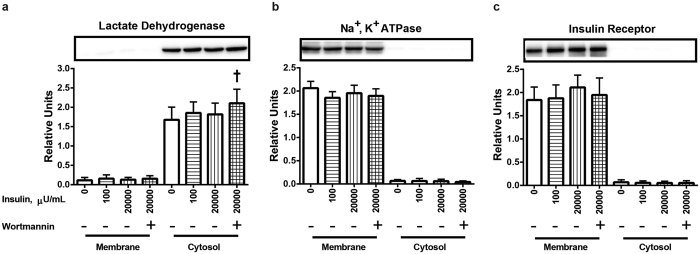
Markers for the cytosol (lactate dehydrogenase) and membranes (Na^+^, K^+^ ATPase and insulin receptor) in the subcellular fractions of muscles. (**a**) Lactate dehydrogenase in membrane and cytosol fractions. (**b**) Na^+^, K^+^ ATPase in membrane and cytosol fractions. (**c**) Insulin receptor in membrane and cytosol fractions. ^†^Significantly (P < 0.05) different from the control group (without insulin or wortmannin). Values are expressed as mean ± SD; n = 10 per treatment.

We used well-recognized markers for the cytosolic (LDH) and membrane (Na^+^, K^+^ ATPase and insulin receptor) fractions to verify that our subcellular fractionation protocol was successful. As expected, the cytosolic fraction was characterized by highly abundant LDH (Fig. 1a) along with essentially undetectable Na^+^, K^+^ ATPase (Fig. 1b) and insulin receptor (Fig. 1c). Conversely, the membrane fraction was enriched with both Na^+^, K^+^ ATPase (Fig. 1b) and insulin receptor (Fig. 1c) together with barely detectable LDH (Fig. 1a). Neither insulin (100 or 20,000 μU/ml) nor wortmannin altered the abundance of Na^+^, K^+^ ATPase or insulin receptor in either fraction. Membrane LDH was also unchanged by insulin or wortmannin. Treating muscles with a combination of insulin and wortmannin resulted in slightly (~25%), but significantly (P < 0.05) greater cytosolic LDH values compared to control muscles that were incubated without insulin and wortmannin. However, insulin alone did not alter the LDH content of the cytosolic fraction. These results demonstrated that our protocol effectively purified the cytosolic and membrane fractions from small (~70 mg) skeletal muscle samples, and indicated that it would be an appropriate protocol to assess insulin’s effects on the subcellular localization of AKT1, AKT2 and AS160 in skeletal muscle.

### Insulin effects on AKT and AS160 localization and phosphorylation

**Figure 2:**
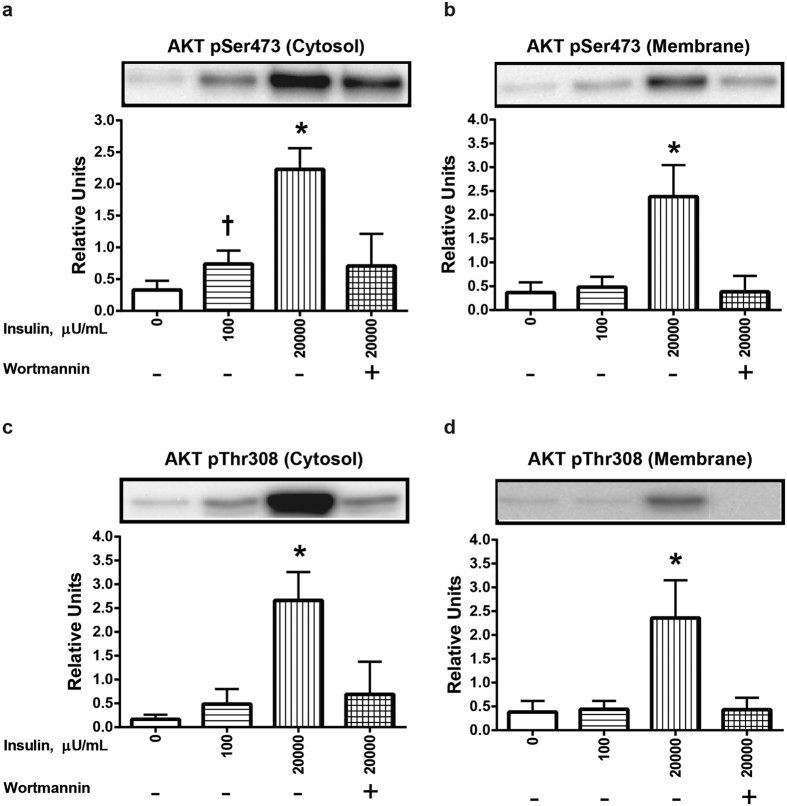
Effects of insulin and wortmannin on phosphorylation of AKT (pAKT) in cytosol and membrane fractions of muscle. (**a**) pAKT Ser473 in the cytosol fraction. (**b**) pAKT Ser473 in the membrane fraction. (**c**) pAKT Thr308 in the cytosol fraction. (**d**) pAKT Thr308 in the membrane fraction. ^†^Significantly (P < 0.05) different from the control group (without insulin or wortmannin). *Significantly (P < 0.05) different from all other groups. There was a non-significant trend (P = 0.056) for increased pAKT Thr308 in the cytosolic fraction of muscle treated with 100 μU/mL insulin. Values are expressed as mean ± SD; n = 10 per treatment.

The AKT antibodies that recognize phospho-threonine (pThr) 308 and phospho-serine (pSer) 473 on AKT1 also recognize pThr309 and pSer474 on AKT2, respectively. In both the cytosolic and membrane fractions, 20,000 μU/mL insulin significantly (P < 0.05) enhanced the level of AKT phosphorylation on Ser473 and Thr308 (P < 0.05) (Fig. 2a–d). A lower insulin concentration (100 μU/mL) also significantly (P < 0.05) increased AKT Ser473 phosphorylation in the cytosolic fraction, but not in the membrane fraction. There was a non-significant trend (P = 0.056) for increased phosphorylation of AKT Thr308 phosphorylation in the cytosolic fraction of muscle treated with 100 μU/mL insulin (Fig. 2c), but not in the membrane fraction. In both the cytosolic and membrane fractions, treatment of insulin-stimulated muscles with the PI3K inhibitor wortmannin eliminated the insulin-induced increase in phosphorylation of AKT on both Ser473 and Thr308.

**Figure 3:**
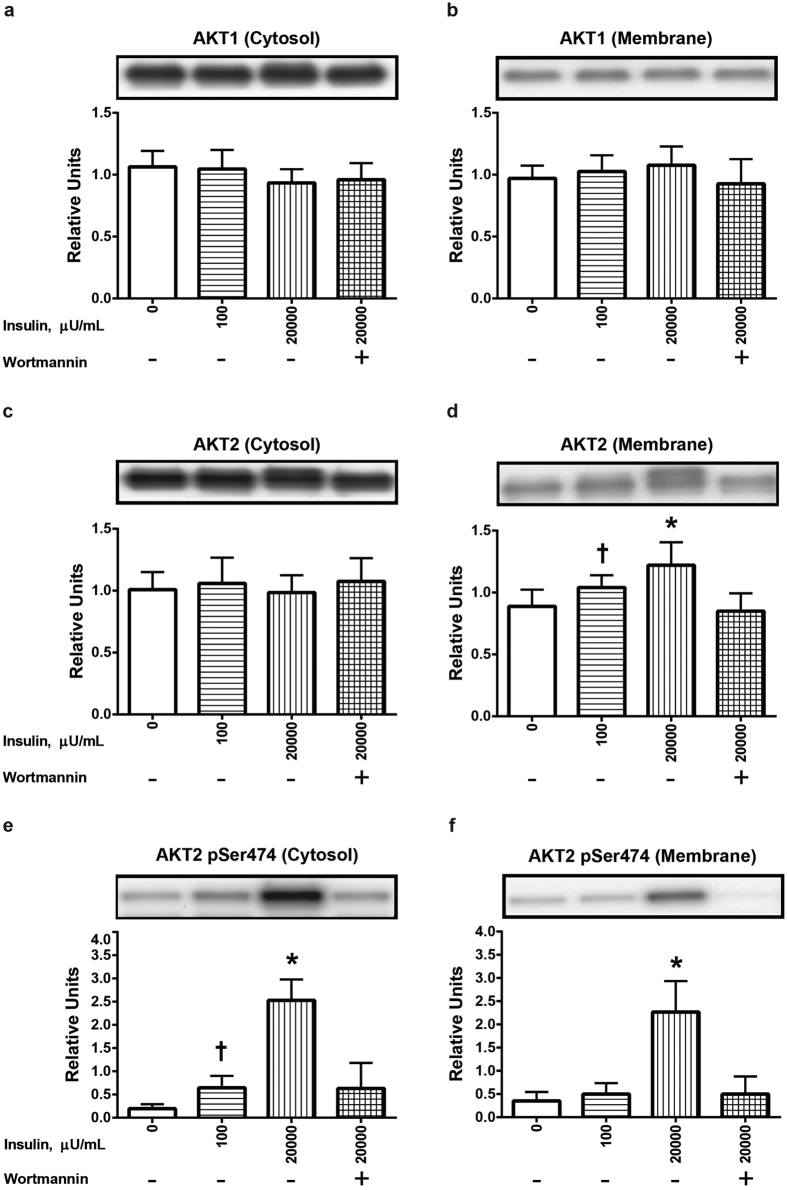
Effects of insulin and wortmannin on subcellular localization of AKT1 and AKT2 and phosphorylation of AKT2 (pAKT2) Ser474 in cytosol or membrane fractions of muscle. (**a**) AKT1 in the cytosol fraction. (**b**) AKT1 in the membrane fraction. (**c**) AKT2 in the cytosol fraction. (**d**) AKT2 in the membrane fraction. (**e**) pAKT2 Ser474 in the cytosol fraction. (**f**) pAKT2 Ser474 in the membrane fraction. ^†^Significantly (P < 0.05) different from the control group (without insulin or wortmannin). *Significantly (P < 0.05) different from all other groups. Values are expressed as mean ± SD; n = 10 per treatment.

Neither insulin (100 or 20,000 μU/mL) nor wortmannin significantly altered AKT1 abundance in either the cytosolic (Fig. 3a) or membrane (Fig. 3b) fraction. AKT2 abundance in the cytosolic fraction was similarly unaffected by insulin or wortmannin (Fig. 3c). In contrast, AKT2 abundance in the membrane fraction was significantly (P < 0.05) increased for muscles incubated with 100 μU/mL insulin compared to muscles incubated without insulin, and membrane accumulation of AKT2 was further increased (P < 0.05) for muscles treated with 20,000 versus 100 μU/mL insulin (Fig. 3d). Furthermore, wortmannin treatment eliminated the significant insulin-induced increase in AKT2 in the membrane fraction. We also evaluated total AKT2 abundance to test if insulin altered AKT2 expression and found total AKT2 abundance did not differ among the treatments (Supplementary Figure 1). Phosphorylation of AKT2 Ser474 was significantly (P < 0.05) increased in the cytosolic fraction of muscles incubated with 100 μU/mL insulin compared to muscles incubated without insulin (Fig. 3e), and 20,000 versus 100 μU/mL insulin caused a further significant (P < 0.05) increase in AKT2 Ser474 phosphorylation in the cytosolic fraction (P < 0.05). In the membrane fraction, 20,000, but not 100 μU/mL insulin, resulted in significantly (P < 0.05) increased AKT2 Ser474 phosphorylation (Fig. 3f). Wortmannin eliminated insulin’s effects on AKT2 Ser474 phosphorylation in both fractions.

**Figure 4:**
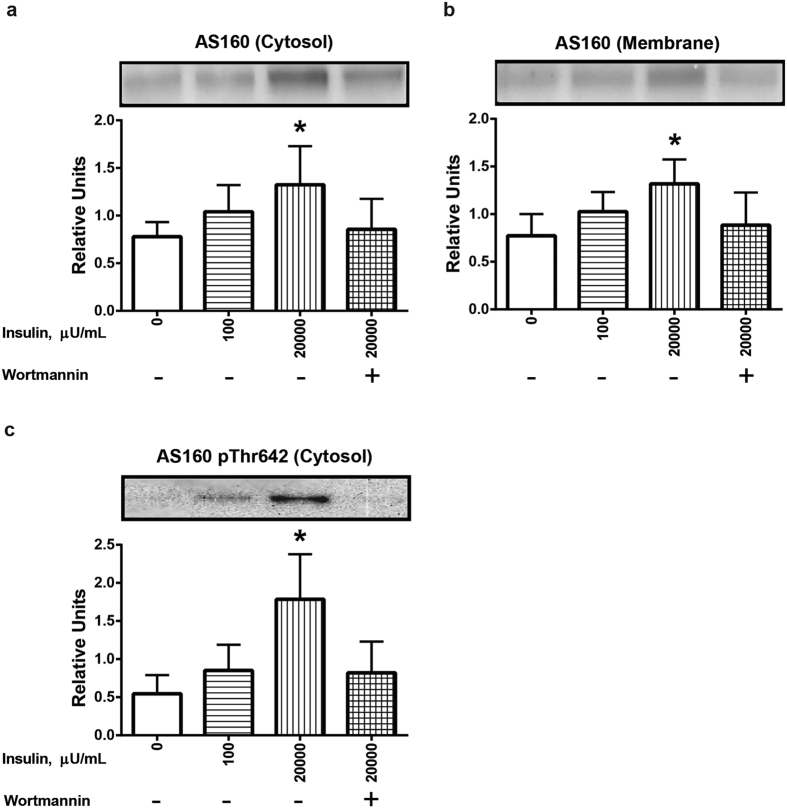
Effects of insulin and wortmannin on subcellular localization and phosphorylation of AS160 (pAS160) Thr642 in cytosol and membrane fractions of muscle. (**a**) AS160 in the cytosol fraction. (**b**) AS160 in the membrane fraction. (**c**) pAS160 Thr642 in the cytosol fraction. There was no detectable pAS160 Thr642 in the membrane fraction, regardless of insulin concentration. *Significantly different from the all other treatment groups (P < 0.05). Values are expressed as mean ± SD; n = 10 per treatment.

**Figure 5:**
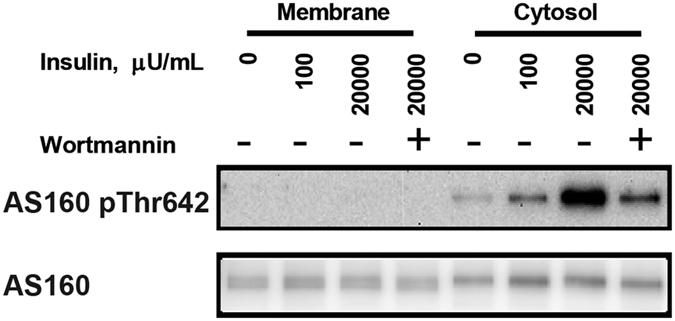
Phosphorylation of AS160 (pThr642) was detectable in the cytosol, but not membrane fraction even though total AS160 abundance was similar between the fractions. Representative blots of AS160 pThr642 and total AS160 for cytosol and membrane fractions from muscles.

Total AS160 abundance in both the cytosolic (Fig. 4a) and membrane (Fig. 4b) fractions was significantly (P < 0.05) increased for muscles incubated with 20,000 μU/mL insulin compared to all other groups. In the cytosolic fraction, AS160 Thr642 phosphorylation of muscles incubated with 20,000 μU/mL insulin exceeded the values for all other treatment groups (Fig. 4c). Wortmannin eliminated the effects of insulin on both AS160 localization in both fractions and on Thr642 phosphorylation in the cytosolic fraction (Fig. 4a–c). In contrast to the robust insulin-stimulated increase in AS160 phosphorylation in the cytosolic fraction, in the membrane fraction, there was no detectable effect of insulin on AS160 Thr642 phosphorylation (Fig. 5). It is important to note that the abundance of total AS160 was roughly similar between the cytosolic and membrane fractions on the same immunoblot. Therefore, the absence of detectable AS160 phosphorylation in the membrane fraction compared to the substantial AS160 phosphorylation in the cytosolic fraction from insulin-stimulated muscles was not attributable to major differences between the fractions in total AS160 levels (Fig. 5).

## Discussion

AKT1 and AKT2 are the AKT isoforms with high expression in skeletal muscle that play key roles to regulate muscle mass and glucose metabolism, respectively^14^,^24^. Insulin’s activation of AKT is crucial for both its anabolic and metabolic actions in skeletal muscle. In adipose cells, insulin differentially alters the subcellular distribution of AKT1 and AKT2, but the current study was apparently the first to assess insulin’s effects on the subcellular localization of both AKT1 and AKT2 in skeletal muscle. The most important new results of this study were that: 1) we validated a rapid and simple method for assessing cytosolic and membrane localized AKT1, AKT2 and AS160 using a small amount of rat skeletal muscle; 2) insulin increased membrane localization of AKT2, but not AKT1 in skeletal muscle; 3) insulin increased AKT2 phosphorylation in both cytosolic and membrane fractions; 4) insulin increased both cytosolic and membrane localization of AS160; 5) insulin increased AS160 phosphorylation in the cytosol, but not membrane fraction; and 6) insulin’s effects on the localization and phosphorylation of AKT2 and AS160 were inhibited by the PI3K-inhibitor wortmannin.

Common methods for evaluating the subcellular localization of proteins include differential centrifugation of cell/tissue lysates and various microscopic methods (e.g., immunohistochemistry, confocal microscopy of fluorescently tagged proteins, and total internal reflectance fluorescence, TIRF, microscopy). Nearly all of the published research on insulin-stimulated effects on AKT1, AKT2 and AS160 localization has used cultured adipocytes. We first validated a suitable method that rapidly separated membrane and cytosolic fractions from relatively small skeletal muscle tissue samples and that did not require access to specialized instrumentation or animals expressing genetically modified proteins. We used a simple differential centrifugation approach that was similar to the procedure originally described by Jacobs *et al*.^25^. The immunblotting of marker proteins verified the method successfully produced membrane and cytosolic fractions from small muscle samples.

Surprisingly few publications have reported the subcellular localization of both AKT1 and AKT2. Moreover, the current study is apparently the first to assess insulin’s influence on the subcellular distribution of both AKT1 and AKT2 in skeletal muscle tissue. In the first published study on the subcellular localization of AKT isoforms, Calera *et al*.^22^ used differential centrifugation to analyze the distribution of AKT1 and AKT2 in adipocytes isolated from rat epididymal fat pads. AKT1 was almost exclusively localized in the cytosol, whereas AKT2 was widely distributed in the cytosol and multiple membrane fractions. However, they did not assess insulin’s effects on AKT1 or AKT2 localization. Sasaoka *et al*.^26^ also used differential centrifugation with 3T3-L1 adipocytes and confirmed that AKT1 was localized in the cytosol under basal conditions. They also demonstrated very little redistribution of AKT1 to membrane fractions upon insulin stimulation. They verified that AKT2 was distributed among cytosolic, plasma membrane and low density membrane fractions under basal conditions and discovered that insulin increased the plasma membrane levels for AKT2, but not AKT1. Gonzalez *et al*.^13^ used quantitative TIRF microscopy to assess subcellular distribution of AKT1 and AKT2 in live 3T3-L1 adipocytes and documented that insulin caused preferentially greater plasma membrane accumulation of AKT2 compared to AKT1. Santi and Lee^23^ used immunohistochemistry with confocal microscopy to visualize the subcellular distribution of AKT1 and AKT2 in a variety of transformed and nontransformed cell lines. AKT1 was primarily localized in the cytosol, whereas AKT2 was largely associated with mitochondria. Using differential centrifugation of MDA-MB231 cells, they confirmed the immunohistochemical results (i.e., AKT1 was mainly localized in the cytosol, and AKT2 was associated with mitochondrial and nuclear fractions). They did not evaluate the influence of insulin on AKT1 or AKT2 localization. The current results were consistent with these earlier studies with regard to insulin’s preferential redistribution of AKT2 to the membrane fraction. Furthermore, our results extended the earlier studies by revealing that insulin’s effects on AKT2 membrane localization: 1) occurs in skeletal muscle, 2) occurs with a physiologic insulin concentration (100 μU/mL); and 3) is wortmannin-inhibitable.

Herr *et al*.^27^ and Yaspelkis *et al*.^28^ used differential centrifugation to assess AKT2, but not AKT1, localization in rat skeletal muscle and reported that insulin (500 μU/mL) did not alter AKT2 abundance in either the cytosol or plasma membrane fractions. Insulin was delivered by hindlimb perfusion, red quadriceps muscles were studied, and the fractionation protocol isolated plasma membranes. We cannot discern if methodological differences account for the discrepancy between these studies and the current study with regard to insulin’s ability to alter AKT2 subcellular localization. However, our results align with previous research using adipocytes that has consistently reported that insulin treatment results in greater AKT2 accumulation in various membrane fractions^13^,^26^.

Multiple studies have used pan-AKT phospho-antibodies that recognize either AKT1/AKT2 Thr308/309 or Ser473/474 in adipocytes^26^,^29^,^30^ to demonstrate that insulin substantially elevates phosphorylation on both sites in both the cytosol and membrane fractions, including plasma membranes, low density microsomes and high density microsomes. The results of the current study also found insulin-stimulated increases in phosphorylation of pan-AKT on both sites in skeletal muscle. In addition, the current results used an AKT2-specific Ser474 antibody to demonstrate that insulin-induced phosphorylation of AKT2 in both the cytosol and membrane fractions. The relative magnitude of the insulin-stimulated increase in the membrane fraction was substantially greater for AKT2 phosphorylation versus AKT2 localization suggesting that insulin’s effects on AKT2 phosphorylation are not only attributable to AKT2 redistribution.

A number of studies have assessed the subcellular localization of AS160 in 3T3-L1 adipocytes^13^,^20^,^21^,^29^,^31^,^32^,^33^,^34^, but the current study is apparently the first to assess AS160’s distribution in skeletal muscle tissue. In adipocytes, results from differential centrifugation indicate that AS160 is detectable in multiple subcellular fractions, including the cytosol and the low and high density microsomes^20^,^21^,^29^,^31^,^32^. A small portion of AS160 co-localizes with GLUT4 storage vesicles^21^,^31^,^33^,^34^. AS160 in plasma membrane fractions has been reported to be either undetectable^20^,^21^,^31^ or barely detectable^29^,^32^. With insulin-stimulation, several studies demonstrated increased cytosolic AS160 abundance^20^,^21^,^29^,^31^,^32^. The results of some^20^,^31^, but not all^29^,^32^ studies suggest that insulin causes reduced AS160 abundance in low density microsomes. The current results for skeletal muscle were consistent with the findings for adipocytes with regard to AS160 being localized in both cytosolic and membrane fractions, and with regard to the insulin-induced increase in cytosolic AS160 abundance. However, the insulin-stimulated increase in AS160 abundance in the membrane fraction of skeletal muscle differed from the observations for adipocytes. It is unclear to what extent the disparate results are attributable to different fractionation protocols and/or differences between muscle tissue and 3T3-L1 adipocytes. The current results also indicated that insulin’s effects on AS160 localization in both the cytosol and membrane fractions were wortmannin-inhibitable.

Research on both adipocytes and skeletal muscle indicates that AKT2 is the primary isoform responsible for insulin-stimulated Thr642 phosphorylation of AS160^12^,^13^,^35^. There was a striking difference between the cytosolic and membrane fractions with regard to AS160 phosphorylation in response to insulin in the current study. AS160 phosphorylation was markedly increased by insulin in the cytosol, but it was undetectable in the membrane fraction. This difference was evident even though there was not a major difference in total AS160 abundance of the cytosol compared to the membrane fraction. These results in muscle differed from earlier observations for 3T3-L1 adipocytes, which were characterized by very little insulin-effect on pAS160 Thr642 in either the cytosol or low density membranes concomitant with substantially increased values in the plasma membrane fraction^29^. The functional significance of this difference remains to be determined.

In conclusion, earlier research on insulin’s regulation of the subcellular distribution of AKT1, AKT2 and AS160 relied exclusively on isolated cells with uncertain relevance to the biology of skeletal muscle, a major insulin target tissue. The current study used a simple method requiring small muscle samples to demonstrate that insulin produces an AKT-isoform selective redistribution of AKT2 along with greater AKT2 phosphorylation in the membranes of skeletal muscle concomitant with elevated AS160 abundance in the cytosol and membrane fractions. The novel insights from this simple procedure offer a new opportunity to elucidate the functional consequences of AKT1, AKT2 and AS160 subcellular localization under conditions of insulin resistance (e.g., obesity, high fat diet, and aging) or improved insulin sensitivity (e.g., exercise or calorie restriction).

## Methods

### Materials

Unless otherwise noted, all chemicals were purchased from Sigma-Aldrich (St. Louis, MO) or Fisher Scientific. Sodium dodecyl sulfate-polyacrylamide electrophoresis apparatus, immunoblotting reagents were obtained from Bio-Rad Laboratories (Hercules, CA). Pierce MemCode Reversible Protein Stain Kit, BCA Protein Assay Kit and Pierce Detergent Compatible Bradford Assay Kit were from Thermo Fisher (Waltham, MA). Anti-phospho-AKT Ser473 (pAKTSer473; #9271), anti-phospho-AKT Thr308 (pAktThr308; #9275), anti-phospho-AKT2 Ser474 (pAKT2Ser474; #8599), anti-phospho-AS160 Thr642 (pAS160Thr642; #8881), anti-AKT1 (# 2938), anti-AKT2 (#3063), anti-Na^+^, K^+^ ATPase (#3010), anti-LDH (#3558), anti-insulin receptor (IR; #3025) and anti-rabbit IgG horseradish peroxidase conjugate (#7074) were from Cell Signaling Technology (Danvers, MA). Anti-AKT substrate of 160 kDa (AS160; ABS54) was obtained from EMD Millipore. Human recombinant insulin was from Eli Lilly (Indianapolis, IN).

### Animal care and treatment

Animal care procedures were approved by the University of Michigan Committee on Use and Care of Animals. All methods were performed in accordance with the guidelines from the Guide for the Care and Use of Laboratory Animals of the National Institutes of Health, USA. Male Wistar rats (weight ~250 g) obtained from Charles Rivers Laboratories (Wilmington, MA) were provided with 5001 laboratory rodent chow (Lab Diet; St. Louis, MO) and water ad libitum until 1700 h on the night before the experiment, when the rats were fasted. The next morning, rats were anesthetized by an intraperitoneal injection of sodium pentobarbital. Both soleus muscles were rapidly extracted and each was longitudinally split into two strips followed by *ex vivo* incubation.

### Muscle incubation

Soleus muscle strips were placed in vials supplemented with appropriate media, shaken at 50 revolutions per minute while continuously gassed (95% O_2_/5% CO_2_) in 35 °C water bath. Muscles were incubated in Krebs-Henseleit buffer (KHB) supplemented with bovine serum albumin (0.1%), sodium pyruvate (2 mM) and mannitol (6 mM). During first step of incubation, three soleus strips from each rat were incubated for 30 min in KHB supplemented with vehicle (0.05% DMSO), and the fourth soleus strip was incubated in KHB supplemented with 500 nM wortmannin (dissolved in vehicle: 0.05% DMSO). During second step of incubation (50 min), three soleus strips were transferred to vials containing KHB and vehicle with 0, 100 or 20,000 μU/mL insulin, and the fourth soleus strip was transferred to a vial containing KHB, vehicle, and 20,000 μU/mL insulin plus 500 nM wortmannin. After the second incubation step, muscles were rapidly blotted on filter paper moistened with ice-cold KHB buffer, trimmed, freeze-clamped by aluminum tongs cooled in liquid nitrogen and stored at −80 °C until subsequent processing.

### Subcellular fractionation

Frozen muscles were weighed and homogenized in 1 mL ice-cold fractionation buffer containing 20 mM Tris-HCl (pH 7.5), 250 mM sucrose, 1 mM EDTA, 1 mM EGTA, 1 mM PMSF, 25 mM NaF, 25 mM β-glycerophosphate, 1 mM Na_3_VO_4_, protease inhibitor cocktail (Sigma, # P8340) using a glass grinding tubes (Kontes, Vineland, NJ) on ice. An aliquot (50 μL) from each homogenate was removed and supplemented with sodium dodecyl sulfate (SDS) to a final SDS concentration of 0.1% and rotated gently for 1 h at 4 °C to solubilize total proteins followed by centrifugation at 15,000 g, 4 °C for 15 min. The resulting supernatant was assayed for protein concentration using the BCA protein assay, and an equal amount of total protein from each sample was used for immunoblotting analysis of total protein as described below. To prepare the subcellular fractions that were used for analysis of protein subcellular localization, the remainder of each homogenate was pre-cleared with centrifugation at 1000 g, 4 °C for 15 min. The pre-cleared supernatant was centrifuged at 135,800 g, 4 °C for 1 h. The resulting supernatant (cytosol fraction) was retained and the pellet (membrane fraction) was washed twice in fractionation buffer without protease inhibitor cocktail and centrifuged at 135,800 g, 4 °C for 10 min. The membrane pellet was then resuspended in fractionation buffer supplemented with 0.1% SDS. Protein concentration was determined by BCA assay for cytosolic fractions or detergent compatible Bradford assay for membrane fractions.

### Immunoblotting

Aliquots from each of the fractions (membrane and cytosol) containing equal amounts of total protein were combined with 4X or 6X protein sample buffer and heated at 95 °C for 2 min. Samples were then subjected to SDS-PAGE and electrophoretically transferred to polyvinyl difluoride (PVDF). Equal loading was confirmed using the MemCode protein stain. Bovine serum albumin (5%) or milk (5%) in Tris-buffered saline, pH 7.5 with 0.1% Tween-20 (TBST) was used for blocking at room temperature for 1 h. PVDF was subsequently incubated with primary antibody diluted with either 5% bovine serum albumin or 5% milk in TBST at 4 °C overnight with gentle rocking. Primary antibody incubation was followed by TBST washes (3 times), before PVDF was incubated with secondary antibody for 1 h at room temperature, followed by TBST washes (3 times) and TBS washes (2 times). Proteins were visualized using enhanced chemiluminescence reagent (Luminata Forte Western HRP substrate, #WBLUF0100; Millipore). Protein bands were quantified by densitometry (AlphaView, ProteinSimple, San Jose, CA). The individual value for each sample was expressed in relative units determined by dividing the densitometry units for each sample by the mean value in densitometry units for all of the samples on the blot.

### Statistical analysis

Statistical analyses were performed using SigmaPlot 13.0 version (Systat Software Inc, San Jose, CA). Data are expressed as the mean ± standard deviation (SD), and P ≤ 0.05 was considered statistically significant. One-way Analysis of Variance (ANOVA) was used to evaluate significant differences among treatments. When data failed the Shapiro-Wilk normality test and/or Brown-Forsythe equal variance test, they were mathematically transformed to attain normality and equal variance. Holm-Sidak pairwise multiple comparisons were performed to identify the source of significant variance.

## Additional Information

**How to cite this article**: Zheng, X. and Cartee, G. D. Insulin-induced Effects on the Subcellular Localization of AKT1, AKT2 and AS160 in Rat Skeletal Muscle. *Sci. Rep.* **6**, 39230; doi: 10.1038/srep39230 (2016).

**Publisher’s note:** Springer Nature remains neutral with regard to jurisdictional claims in published maps and institutional affiliations.

## Supplementary Material

Supplementary Figure 1 and Legend
