# Supplementary material for: Insulin-induced Effects on the Subcellular Localization of AKT1, AKT2 and AS160 in Rat Skeletal Muscle
Source: Sci Rep. 2016 Dec 14;6:39230. doi: 10.1038/srep39230 (PMC5155274; doi:10.1038/srep39230)
Supplement: Supplementary Figure 1 and Legend [file srep39230-s1.pdf]

Insulin-induced Effects on the Subcellular Localization of AKT1, AKT2 and AS160 in Rat Skeletal Muscle

Xiaohua Zheng and Gregory D. Cartee

Supplementary

Figure 1. *Effects of insulin and wortmannin on total AKT2 abundance in muscle.* Values are expressed as mean ±SD; n = 10 per treatment.

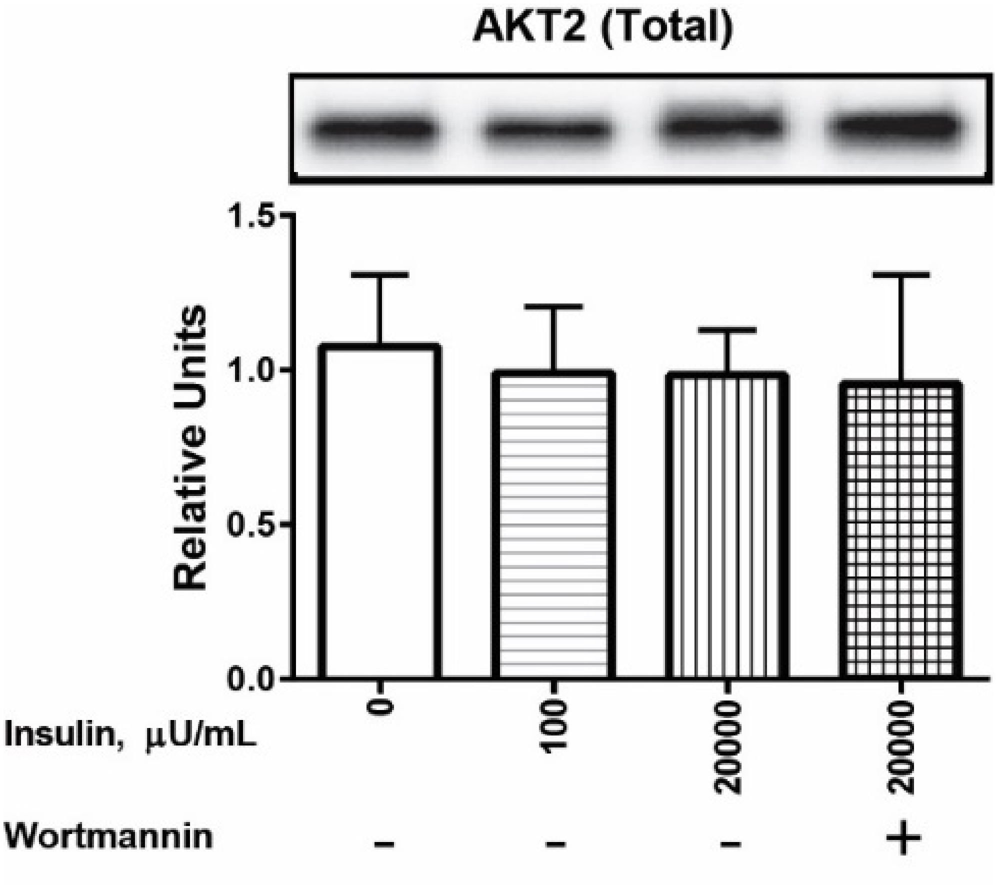
AKT2 (Total)
1.5
1.0
0.5
0.0
Relative Units
Insulin, μU/mL
0
100
20000
20000
Wortmannin
-
-
-
+
